# Preparation of Transparent and Thick CNF/Epoxy Composites by Controlling the Properties of Cellulose Nanofibrils

**DOI:** 10.3390/nano10040625

**Published:** 2020-03-28

**Authors:** Shin Young Park, Simyub Yook, Sooim Goo, Wanhee Im, Hye Jung Youn

**Affiliations:** 1 Department of Forest Sciences, College of Agriculture and Life Sciences, Seoul National University, Seoul 08826, Korea; sypk1992@snu.ac.kr (S.Y.P.); breedingcrops@snu.ac.kr (S.Y.); ksi960@snu.ac.kr (S.G.); 2 Research Institute of Agriculture and Life Sciences, Seoul National University, Seoul 08826, Korea; cimjjang@gmail.com

**Keywords:** carboxymethylation, cellulose nanofibrils, composite, solvent exchange, transparency

## Abstract

Cellulose nanofibrils (CNFs) have been used as reinforcing elements in optically transparent composites by combination with polymer matrices. In this study, strong, optically transparent, and thick CNF/epoxy composites were prepared by immersing two or four layers of CNF sheets in epoxy resin. The morphology of the CNF, the preparation conditions of the CNF sheet, and the grammage and layer numbers of the CNF sheets were controlled. The solvent-exchanged CNF sheets resulted in the production of a composite with high transparency and low haze. The CNF with smaller width and less aggregated fibrils, which are achieved by carboxymethylation, and a high number of grinding passes are beneficial in the production of optically transparent CNF/epoxy composites. Both the grammage and number of stacked layers of sheets in a composite affected the optical and mechanical properties of the composite. A composite with a thickness of 450–800 μm was prepared by stacking two or four layers of CNF sheets in epoxy resin. As the number of stacked sheets increased, light transmittance was reduced and the haze increased. The CNF/epoxy composites with two layers of low grammage (20 g/m^2^) sheets exhibited high light transmittance (>90%) and low haze (<5%). In addition, the composites with the low grammage sheet had higher tensile strength and elastic modulus compared with neat epoxy and those with high grammage sheets.

## 1. Introduction

The use of petroleum-based plastics has caused serious environmental problems, increasing the need for alternative eco-friendly materials. Diverse bio-resources, including wood, grass, and algae, have been used for producing new sustainable materials. Among them, cellulose nanofibrils (CNFs) have recently been vaunted as a promising eco-friendly material. CNFs are nano-sized cellulose materials of a few nanometers in width and a few micrometers in length. CNFs can be obtained by the mechanical treatment of pulp fibers with or without mechanical, chemical, or biological pretreatment [1,2,3]. Owing to their high strength, thermal stability, large surface specific area, and biodegradability, CNFs have been used as sustainable reinforcing elements in various matrices, including thermoplastics [4,5,6,7,8], bioplastics [9,10], and rubber [11,12].

Because CNFs are a few nanometers in width, the light scattering of the CNF-containing materials can be reduced [13,14,15,16]. Many studies have reported that CNF can be used to produce optically transparent composites by itself or by mixing with other transparent polymers [17,18,19,20,21,22,23]. Optically transparent CNF composites are expected to be applied in packaging, display, optoelectronics, or automobiles by replacing glass or plastics owing to its high transparency, outstanding mechanical properties, greater safety, and sustainability. Within the previous studies, the preparation of the composites by mixing CNF with transparent resins such as acrylic resin or epoxy resin has drawn much interest [18,21,24,25,26]. As these resins have a similar refractive index to that of CNF, the light scattering of the composites could be minimized [13,24]. CNF/epoxy composites can be simply manufactured by coating and impregnation of CNF sheet with epoxy resin, and mixing and extrusion of the CNF and epoxy resin. In the case of coating of the CNF sheet with epoxy resin [26,27], the resin covers the surface and smoothens the roughness of the CNF sheet, which increases the transparency of the composite [26]. In addition, the CNF/epoxy composite is prepared by immersing the CNF sheet into the epoxy resin [18,24]. The resin penetrates into the sheet and can reduce the light scattering of the CNF sheet [13,20].

Although many studies have revealed that CNF and transparent resin can form reinforced transparent composites, most have focused on the improvement of the mechanical strength of the composite. The composites studied had relatively thin thicknesses below 200 μm, which is only useful in limited applications. In addition, a detailed investigation of the relationship between CNF properties and the optical properties of composites is required. Therefore, this study aimed to prepare thick and optically transparent CNF/epoxy composites by controlling the properties of the CNF and CNF sheets. The CNF/epoxy composites were prepared by impregnating CNF sheets with epoxy resin. During preparation, several factors, including the CNF morphology, the CNF sheet preparation method, and the grammage and number of the CNF sheets, were controlled. The effects of the properties of the CNF and CNF sheets on the characteristics of the composites were investigated in terms of light transmittance, haze, and tensile properties. CNF morphology was controlled by carboxymethylation pretreatment and the number of grinding passes. By comparing the dried CNF sheet and the solvent-exchanged sheet during the immersion process, the effects of the miscibility and porosity of the CNF sheet on optical properties of the composite were investigated. Through this study, the key factors in preparing thick, strong, and transparent CNF/epoxy composites were determined.

## 2. Materials and Methods

### 2.1. Raw Materials

Hardwood bleached kraft pulp (HwBKP) for CNF preparation was supplied by Moorim P&P (Ulsan, Korea). For the carboxymethylation process, monochloroacetic acid (Sigma Aldrich, St. Louis, MO, USA), sodium hydroxide, and isopropanol (Samchun Chemicals Co. Ltd., Seoul, Korea) were used. Epoxy resin (KDS 8128, Kukdo Chemical, Seoul, Korea) and a hardener (MH 700G, New Japan Chemical Co. Ltd., Osaka, Japan) were used for the preparation of the composite. 2,4,6-Tris(dimethylaminomethyl)phenol (Sigma Aldrich) was used as a catalyst for epoxy hardening.

### 2.2. Preparation of Untreated CNF and Carboxymethylated CNF Sheets

#### 2.2.1. Carboxymethylation of Pulp

Carboxymethylation of HwBKP as a pretreatment for CNF production was conducted according to the method described by Im et al. [28]. The pulp (dry weight of 80 g) was added to a 2 L solution of sodium hydroxide (NaOH) in isopropanol and reacted at 35 °C for 30 min. Next, monochloroacetic acid was added to the suspension. To control the carboxyl content of the carboxymethylated pulp, the quantity of monochloroacetic acid added was controlled at 0.25 to 1 mmol/g based on the oven-dried weight of the pulp. The pulp was then reacted at 65 °C for 1 h. Following the reaction, the pulp was adequately washed with deionized water. The carboxyl group content of each pulp was measured by a conductometric titration method in accordance with SCAN-CM 65:02 (total acidic group content).

#### 2.2.2. Preparation of Untreated CNF and Carboxymethylated CNF

Untreated CNF (U-CNF) and carboxymethylated CNF (CM-CNF) were prepared by grinding untreated and carboxymethylated pulp suspensions, respectively, using a Supermasscolloider (Masuko Sangyo, Kawaguchi, Japan). The U-CNF was prepared by grinding a 1.5% pulp suspension 24 times. In the case of CM-CNF, the carboxymethylated pulp suspension passed through the grinder 4 times and 10 times, respectively, to investigate the effect of the grinder pass number on the properties of the composite. The rotational speed and gap size during the grinding process were 1500 rpm and −80 μm, respectively. A scanning electron microscope (SEM, SUPRA 55VP, Carl Zeiss, Oberkochen, Germany) was used to examine the morphology of the CNF. A diluted CNF suspension (0.005%) dropped onto Cu grid was dried under room temperature and coated by Pt with 5 nm thickness, and the observation was conducted at 2 kV. SEM images were used for measuring the fibril width using Image J program. As the CNF samples were coated by Pt for SEM observation, the width of fibrils can be affected by coating thickness. The fibril width would be larger under SEM measurement compared with measurement using atomic force microscope or transmission electron microscope. Pt coating was conducted in the same manner for all kinds of CNFs, however, the effect of coating thickness was ignored in this work. The average width and width distribution were obtained from the measurements on at least 100 fibrils for each kind of CNF.

#### 2.2.3. Preparation of Dried- and Solvent-Exchanged-CNF Sheets

To evaluate the effect of miscibility and porosity of the sheet on the optical properties of the composite, the dried sheets were prepared using the U-CNF, and solvent-exchanged wet sheets were prepared using both the U-CNF and CM-CNF suspensions, respectively. For the dried CNF sheet, 0.5% CNF suspension was de-watered on a mixed cellulose ester membrane filter (Advantec, Vernon Hills, IL, USA) by vacuum filtration. After filtration, the sheet was pressed under a pressure of 30 bar for 5 min and hot-pressed at 105 °C and 25 bar for 15 min. For solvent-exchanged sheets, 0.5% suspension was filtrated by vacuum, and the wet sheet was pressed at 30 bar for 10 s. The sheet was then immersed in an ethanol solvent for more than 12 h. The solvent-exchanged sheet was pressed for 10 s, and then used for composite preparation without drying. To investigate the effect of sheet grammage on the properties of the composite, solvent-exchanged CM-CNF (550 μmol/g) sheets were prepared with grammage values of 20, 30, and 40 g/m^2^, respectively, by adjusting the amount of CM-CNF suspension.

### 2.3. Preparation of CNF/Epoxy Composite

**Scheme 1:**
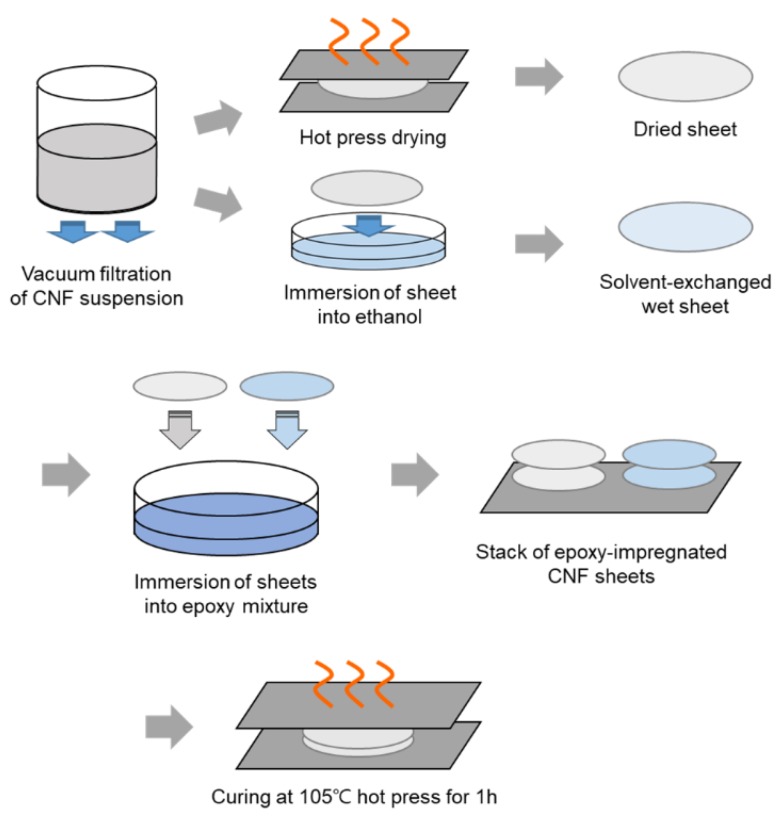
Overall process for the preparation of cellulose nanofibril (CNF)/epoxy composites.

The epoxy resin, hardener, and catalyst were mixed in a weight ratio of 100:50:0.1 and degassed in a vacuum oven. Each CNF sheet was immersed in the epoxy resin mixture for 30 s until the resin-penetrated sheet became transparent. After removing the impregnated CNF sheet and eliminating the excessive resin from its surface, two or four layers of the resin-impregnated sheets were stacked to produce a thick composite. Then, the sheets were cured at 105 °C and 30 bar in a hot press for 1 h. The overall process for the preparation of CNF/epoxy composites is shown in Scheme 1.

### 2.4. Characterization of the CNF/Epoxy Composite

The optical properties of the CNF/epoxy composite were evaluated using a UV-Vis (ultraviolet-visible) spectrophotometer (Cary 100, Agilent, Santa Clara, CA, USA). The light transmittance of each composite was measured over the wavelength of visible light from 400 to 800 nm. Haze was measured in accordance with ASTM-D1003-11 (Standard Test Method for Haze and Luminous Transmittance of Transparent Plastics), and four values (*T*_1_–*T*_4_) were measured for each sample. *T*_1_ represents only incident light intensity with reflectance standard, and *T*_2_ represents the total light transmitted by a specimen. *T*_3_ is the intensity of the light scattered by the instrument, and *T*_4_ is the intensity of the light scattered by both instrument and specimen. From these values, the total luminous transmittance (*T_t_*), diffuse transmittance (*T_d_*), and haze were calculated using Cary WinUV Color Application software (Agilent, Santa Clara, CA, USA), according to the following equations:

(1) $$T_{t}=T_{2}/T_{1}$$

(2) $$T_{d}=\left[T_{4}-T_{3}\left(T_{2}/T_{1}\right)\right]/T_{1}$$

(3) $$\mathrm{Haze}\;\left(\%\right)=T_{d}/T_{t}\times 100$$

The tensile properties of the composite were evaluated using a universal testing machine (Instron, Norwood, MA, USA). The composite samples were cut into specimens of 50 mm length and 5 mm width. Tests were performed at 20 mm span length, and the strain speed was 20 mm/min. More than five specimens for each sample were measured.

## 3. Results and Discussion

### 3.1. Effect of Solvent-Exchange Process of the Sheet on the Composites Properties

**Figure 1:**
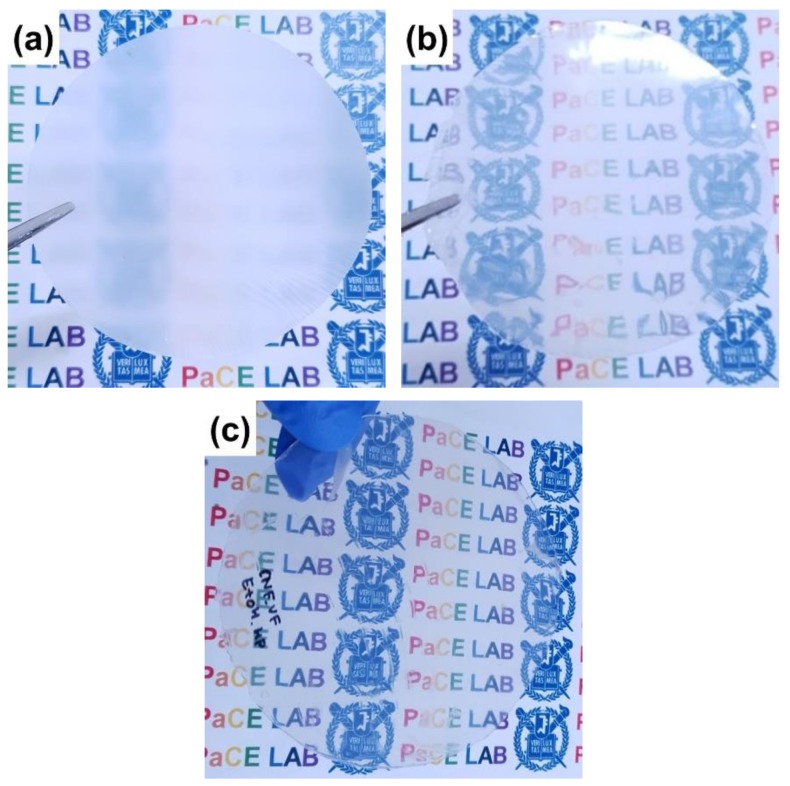
Appearance of (**a**) a single untreated cellulose nanofibril (U-CNF) sheet, (**b**) a two-layered dried U-CNF/epoxy composite, and (**c**) a two-layered solvent-exchanged U-CNF/epoxy composite. Pictures were taken at a distance of a few centimeters from the background printed paper.

**Figure 2:**
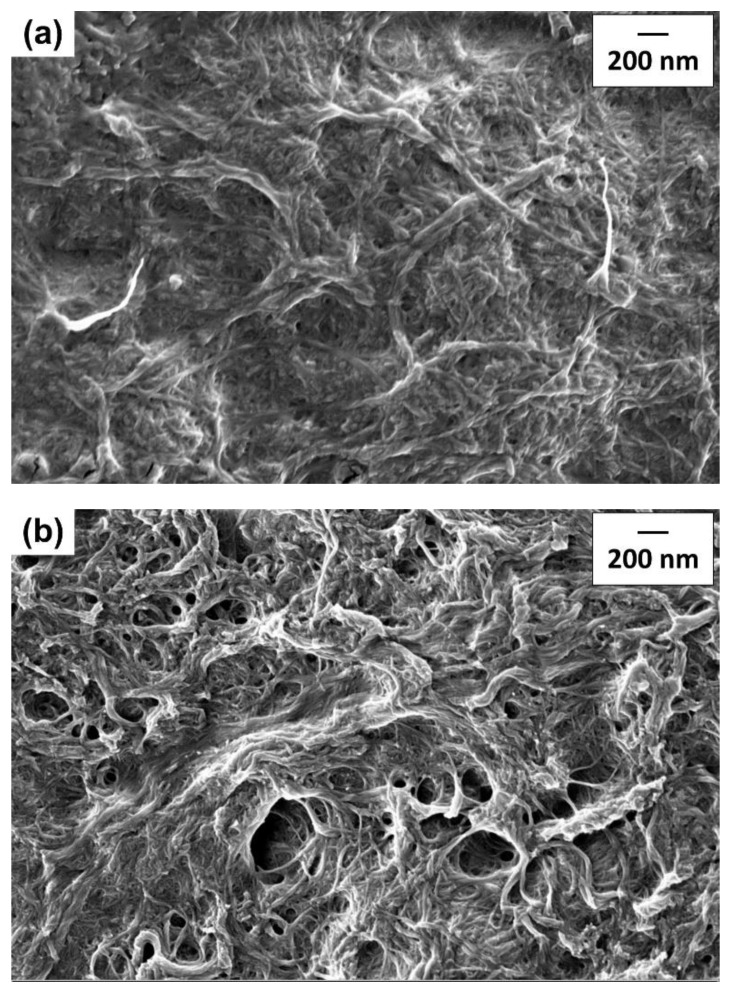
Scanning electron microscope (SEM) images of (**a**) the dried (hot-pressed) U-CNF sheet and (**b**) the solvent-exchanged U-CNF sheet. Image of the solvent-exchanged sheet was taken after air-drying.

Composites of U-CNF sheets and epoxy were prepared by stacking two layers of dried and solvent-exchanged U-CNF sheets, which were immersed into epoxy resin, respectively. The grammage of each sheet was 20 g/m^2^. Figure 1 shows the U-CNF sheet (single layer) before epoxy immersion and U-CNF/epoxy composites prepared with two-layer dried sheets and the solvent-exchanged sheets. Compared with the U-CNF single sheet, the two-layered U-CNF sheet/epoxy composites became transparent. In particular, the composite prepared with the solvent-exchanged sheet demonstrated a higher transparency than that with the dried sheet. It is notable that two-layered U-CNF sheet/epoxy composites look more transparent than the single U-CNF sheet, even though the composites had larger thickness. The penetration of epoxy resin into the sheets contributed to improving the transparency of composites. Table 1 presents the optical properties of the U-CNF sheet and U-CNF/epoxy composites. The dried U-CNF sheet had light transmittances close to 90% and 76.0% of haze. Although the U-CNF sheet had high transmittance, it appeared translucent because of a high haze value. When the U-CNF sheets were impregnated with epoxy, U-CNF/epoxy composites appeared more transparent, despite having a lower transmittance than U-CNF sheets. This may be attributed to a lower haze value, because epoxy resin penetrated into the sheets. As epoxy and CNF have a similar refractive index, the epoxy-impregnated CNF sheets exhibited low light scattering, which leads to a higher transparency [13,20,26]. In addition, the haze of the composite was further reduced when prepared with the solvent-exchanged sheets. This can be explained by two reasons, one of which is the improved miscibility between the CNF and epoxy matrix. CNF is hydrophilic polymer, and mixing with a hydrophobic material is difficult. However, solvent-exchange treatment decreases the hydrophilicity of the CNF, which results in good compatibility between the fiber and matrix. The second reason is that solvent-exchanged CNF sheets could be immersed in epoxy resin without drying. This means that the solvent-exchanged CNF sheet could avoid the aggregation of fibrils or the formation of hydrogen bonding between fibrils, which can occur during the drying process. Thus, the structure of the solvent-exchanged CNF sheet became more porous and less packed compared with that of the dried CNF sheet (Figure 2). This open structure of the solvent-exchanged CNF sheet could contribute to easier penetration of epoxy resin into the sheet, and ultimately result in higher transparency of the composite [25].

### 3.2. Effect of CNF Morphology on the Composite Properties

**Figure 3:**
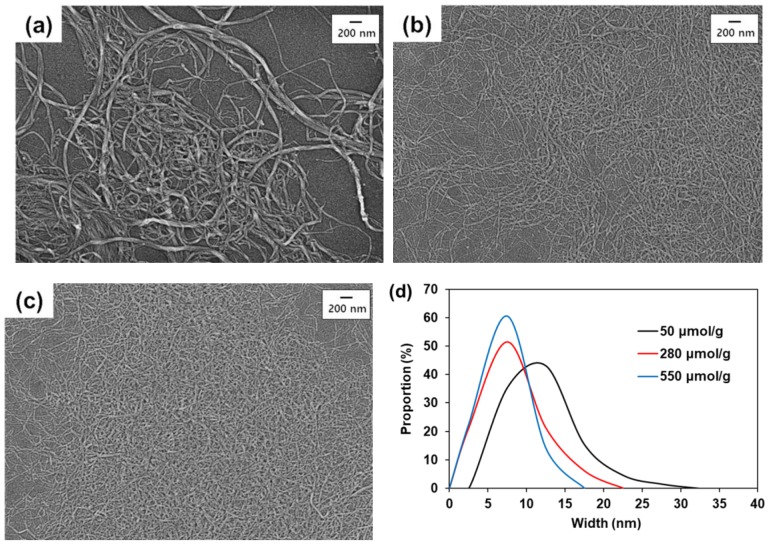
SEM images of (**a**) U-CNF (50 μmol/g carboxyl content), (**b**) carboxymethylated (CM)-CNF with 280 μmol/g carboxyl content, (**c**) CM-CNF with 550 μmol/g carboxyl content, and (**d**) fibril width distribution of CNF with various carboxyl contents.

To evaluate the effect of the carboxyl group content of CNF on the optical property of CNF/epoxy composites, U-CNF and CM-CNF sheets with different carboxyl group contents were used for composite preparation. U-CNF had a carboxyl group content of 50 μmol/g, which was originated from hemicelluloses or created during the pulping and bleaching processes [28,29]. All CM-CNFs used in this section were prepared by passing the grinder four times. The grammage of each CNF sheet was 20 g/m^2^. The composites were prepared by stacking two layers of solvent-exchanged sheets after immersion in the epoxy resin. The morphology of each CNF with different carboxyl contents is shown in Figure 3. As the carboxyl content of the CNF increased, the average fibril width decreased. The carboxyl group content of the pulp affected the morphology of the CNF. It is known that pulp fibers with a higher carboxyl content result in CNF with a more uniform and narrower width compared with untreated or lower carboxyl content fibers [28]. The optical properties of the composites with these CNFs are presented in Table 2. The composite prepared with a high carboxyl content CM-CNF demonstrated the highest light transmittances near 90%, and the lowest haze value at approximately 10%. According to previous studies, the size of fibrils greatly affects the light scattering properties of the CNF sheet. An increase in fibril width results in an increase in forward light scattering and leads to the formation of optically hazed CNF sheets [20,30,31]. Thus, a higher carboxyl content in the CNF results in an improvement in optical properties, that is, higher light transmittance and lower haze.

**Figure 4:**
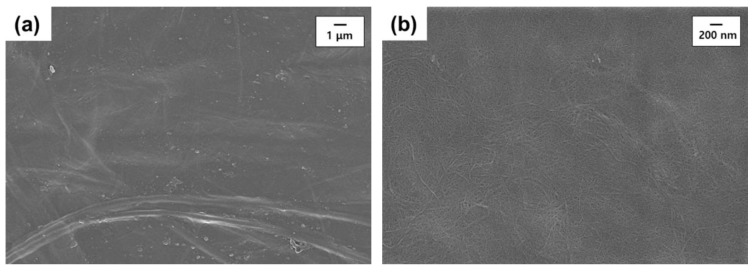
SEM images of CM-CNF sheets (550 μmol/g carboxyl group content): (**a**) 4 passes and (**b**) 10 passes through the grinder.

**Figure 5:**
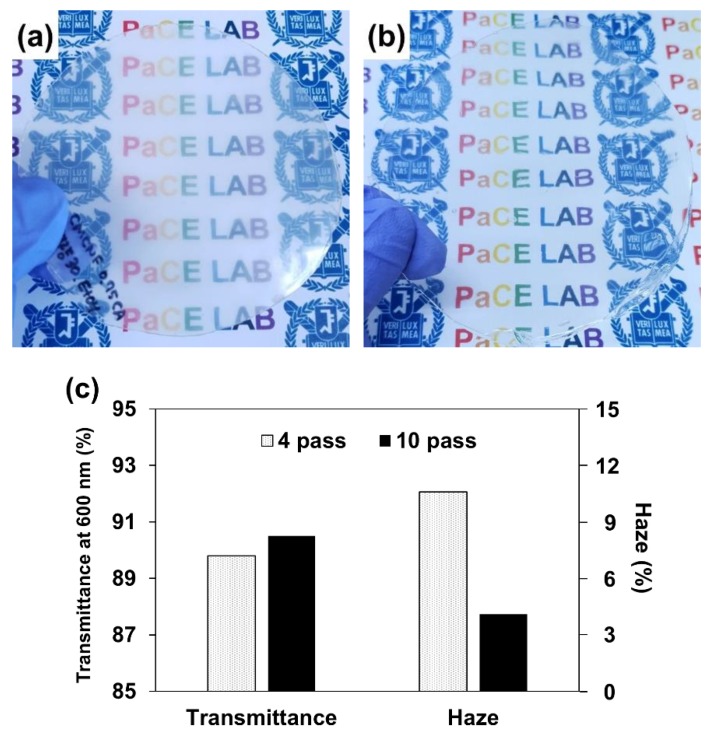
Appearances and optical properties of two-layered solvent-exchanged CM-CNF sheet/epoxy composites: (**a**) composite with four-pass CM-CNF, (**b**) composite with 10-pass CM-CNF, and (**c**) light transmittance and haze of each composite. The carboxyl content of the CM-CNF was 550 μmol/g and each CM-CNF sheet had a grammage of 20 g/m^2^.

CNF morphology can also be controlled by the number of passes through a grinder. To investigate the effect of grinding pass numbers of the CM-CNF on the transparency of the CM-CNF/epoxy composite, CM-CNF (550 μmol/g carboxyl content) was ground 4 times and 10 times, respectively, using a grinder. The SEM images of 4 and 10 passes of the CM-CNF are shown in Figure 4. After passing through the grinder four times, most fibers were nanofibrillated, but some micro-sized fibrils were observed. Conversely, after grinding 10 times, micro-size fibrils were not observed, and all fibers were deconstructed to nanofibrils with nano-scale widths. The two-layered CM-CNF/epoxy composites were prepared using these CM-CNF sheets (20 g/m^2^ for each sheet) after solvent exchange. The optical properties of the CM-CNF/epoxy composite are presented in Figure 5. Higher pass numbers correlated to increasing transparency of the composite. The light transmittance increased slightly from 89.8% to 90.9%, and the haze decreased significantly from 10.6% to 4.1%. As mentioned above, fibrillar size is a crucial factor for the transparency of the composite [20,30]. Because some microfibrils remained after four passes through the grinder, relatively high light scattering occurred throughout the composite, increasing its haze. However, in the case of 10 passes, micro-size fibrils disappeared by complete nanofibrillation, which led to an increase in light transmittance and a decrease in the haze. Therefore, this indicates that the morphology of CNF is an important factor affecting the optical properties of the composite, particularly its haze.

### 3.3. Effects of Sheet Grammage and the Number of Sheet Layers on the Composite Properties

**Figure 6:**
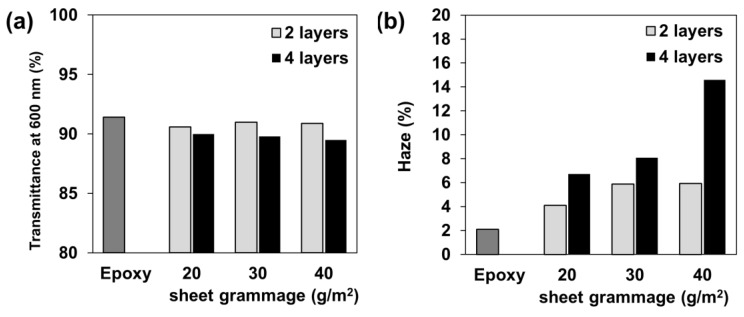
The (**a**) light transmittance at 600 nm and (**b**) haze of neat epoxy and CM-CNF/epoxy composites. The carboxyl content of the CM-CNF was 550 μmol/g.

The effects of the grammage of each CM-CNF sheet and the number of stacked layers in the composites on the optical and physical properties of the composite were investigated. These experiments could determine the proper conditions for the preparation of highly transparent, thick, and strong CM-CNF/epoxy composites. The solvent-exchanged CM-CNF (carboxyl content of 550 μmol/g) sheet was used for the preparation of composites. The optical properties of solvent-exchanged CM-CNF sheet/epoxy composites with different sheet grammage values and numbers of sheet layers are shown in Figure 6. The CM-CNF/epoxy composites exhibited similar light transmittance (above 90%) and higher haze values compared with neat epoxy. There was an increase in the composite’s haze that corresponded with the increased sheet grammage. This may result from increased CNF-epoxy interface area, or porosity formation as a result of incomplete epoxy penetration into the higher grammage CNF sheets. For the layered composites, as the number of stacked sheets increased, light transmittance was reduced and the haze increased. This may be expected to occur if the interfaces between the CNF sheets are imperfect (e.g., having larger defects, delamination, porosity), which would increase light scattering. An increase in thickness of the CNF sheet was likely to be one of factors increasing the haze value. However, an increase in the interfaces appeared to be a more important factor to the haze. Thus, in terms of the transparency of the composite, it appears that an increase in the grammage of sheets is less detrimental than increasing the number of stacked layers.

**Figure 7:**
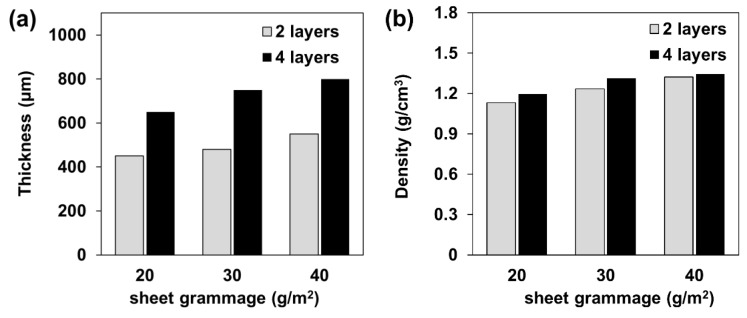
The (**a**) thickness and (**b**) density of each CM-CNF/epoxy composite with different sheet grammage values and numbers of sheet layers.

The thickness and density of each composite are shown in Figure 7. As the grammage and number of layers of the sheets increased, the thickness of the composite also increased. However, the thickness of the composite was not proportional to the layer number and grammage of the sheet. When the sheets were stacked in two layers, the composites were approximately 450–550 μm thick. Those with four layered sheets had a thickness of 650–800 μm. Thus, a thicker composite could be prepared by stacking the CM-CNF sheets. In the case of the composite’s density, higher sheet grammage and sheet layers led to an increase in density.

**Figure 8:**
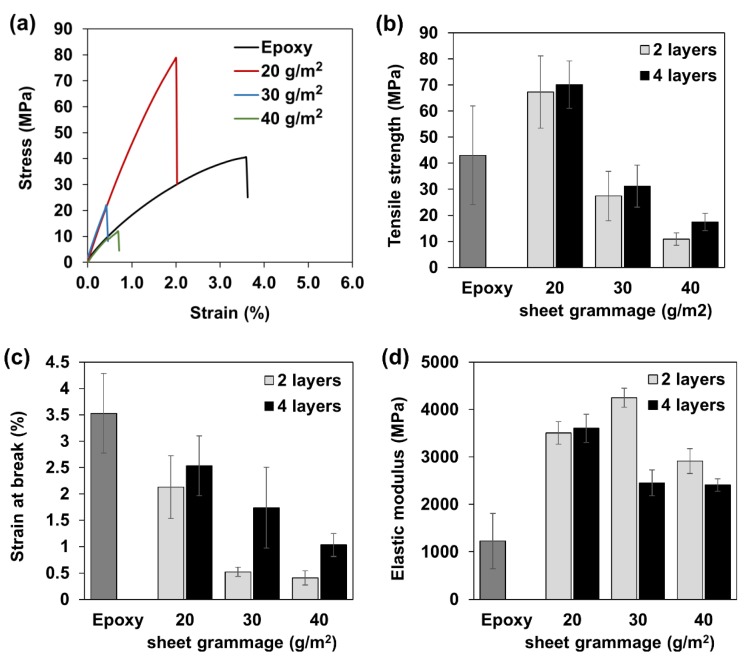
Mechanical properties of multilayered CM-CNF/epoxy composites: (**a**) strain–stress curve of the composites with two layers of CM-CNF sheets, (**b**) tensile strength, (**c**) strain at break, and (**d**) elastic modulus.

**Figure 9:**
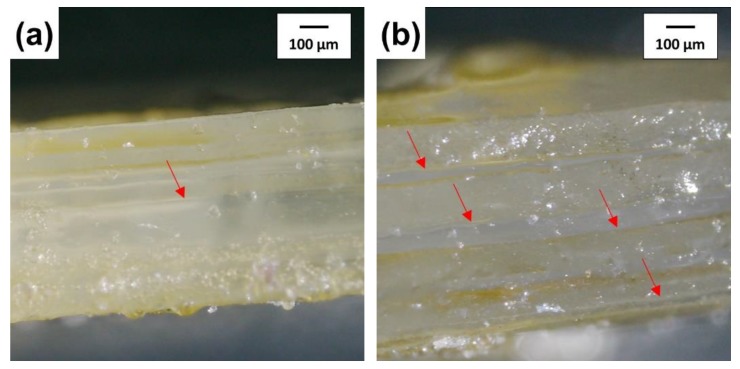
Cross-sectional images of CM-CNF/epoxy composites with (**a**) four layers of 20 g/m^2^ sheets and (**b**) four layers of 40 g/m^2^ sheets (red arrows indicate the interfaces between the CM-CNF sheet layer and epoxy layer).

The tensile properties of the composites are shown in Figure 8. The tensile strength of the composites made from 20 g/m^2^ sheets was 1.5 times greater than neat epoxy. Additionally, the CM-CNF/epoxy composites exhibited a lower breaking strain than neat epoxy. The elastic modulus of the composite increased three times higher than that of neat epoxy because of the effect of the CNF [18,26]. The four layers of sheets led to a further increase in strength compared with the two-layer sheet composites. However, as the sheet grammage increased from 20 to 40 g/m^2^, the composite became weak and brittle, and the breaking strain decreased. The tensile strength of the composite prepared using 40 g/m^2^ sheets decreased severely, and the elastic modulus was hard to determine as they were quickly broken. The effect of CNF sheets on mechanical properties of CNF/epoxy composites could be explained by the amount of CNF in the composite, and the penetration and adhesion of epoxy resin into the CNF sheet [26,32]. Saba et al. [32] prepared a CNF/epoxy composite by adding freeze-dried CNF powder into epoxy resin and reported that the tensile properties of the CNF/epoxy composite were increased by CNF loading up to a certain content, and then decreased owing to inhomogeneous dispersion of CNF in the epoxy matrix. Although the composite preparation process was different from the literature, it revealed that the reinforcing effect of CNF seemed to diminish with the increasing amount of CNF in the composite. The weight portions of the CNF sheet were 8% and 16% for lower-grammage (20 g/m^2^) and higher-grammage (40 g/m^2^) sheets, respectively. Figure 9 shows cross-sectional images of the four-layered composites with sheets of different grammage values. For lower-grammage sheets, it was observed that resin could penetrate deeper into the sheet. Therefore, the interfaces (red arrow) between the CNF sheet layers and the epoxy layers were hard to distinguish in the cross-sectional image of the composite (Figure 9a). On the contrary, it appears that epoxy resin could not penetrate deeply into the higher-grammage sheet as distinct layers of CNF sheets can be observed in the cross-sectional image (Figure 9b). Therefore, the different penetration behaviors of epoxy resin into CNF sheets might cause differences in the mechanical properties of the composites.

## 4. Conclusions

The CNF/epoxy composites in this study were prepared by impregnating epoxy resin into the CNF sheets, which differed in morphology, sheet preparation methods, sheet grammage, and number of sheet layers. Composite properties were evaluated in terms of light transmittance, haze, and tensile properties. It was revealed that solvent exchange of the CNF sheet could improve the transparency of the composite by reducing a haze. The CNF morphology was shown to affect the light transmittance and haze properties of the composite. The smaller fibrils and no-fibril bundles, which were obtained by carboxymethylation and high numbers of grinding passes, produced a composite with high transmittance and low haze. Low CM-CNF sheet grammage (20 g/m^2^) resulted in the composite with a higher light transmittance (>90%) and low haze value (<5%), and also demonstrated a reinforcing effect for the composite. However, a higher sheet grammage value resulted in decreased tensile properties and lower transparency of the composite. As the number of sheet layers increased, the tensile strength and thickness of the composite increased, whereas the transparency decreased owing to an increase in the haze of the composite. Consequently, a thick CM-CNF/epoxy composite with improved mechanical properties without a severe decrease in transparency was prepared by immersing solvent-exchanged low-grammage CM-CNF sheets with narrow and uniform fibrils into epoxy resin and stacking them.

## Figures and Tables

**Table 1:** Optical properties of the untreated cellulose nanofibril (U-CNF) sheet and the U-CNF/epoxy composites.

Sample	Transmittance at 600 nm (%)	Haze (%)
Dried U-CNF sheet (single layer)	89.6	76.0
Two-layered dried U-CNF/epoxy composite	83.9	30.4
Two-layered solvent-exchanged U-CNF/epoxy composite	83.9	26.2

**Table 2:** Optical properties of two-layered solvent-exchanged CNF sheet/epoxy composites with different carboxyl contents of CNF. CM-CNF, carboxymethylated CNF.

CNF Type	Carboxyl contents of CNF (μmol/g)	Transmittance at 600 nm (%)	Haze (%)
U-CNF	50	83.9	26.2
CM-CNF	280	84.6	21.1
	355	86.3	17.2
	470	88.3	15.0
	550	89.8	10.6

## References

[1] Kim J.-H., Shim B.S., Kim H.S., Lee Y.-J., Min S.-K., Jang D., Abas Z., Kim J. (2015). Review of nanocellulose for sustainable future materials. Int. J. Precis. Eng. Manuf. Green Technol..

[2] Abitbol T., Rivkin A., Cao Y., Nevo Y., Abraham E., Ben-Shalom T., Lapidot S., Shoseyov O. (2016). Nanocellulose, a tiny fiber with huge applications. Curr. Opin. Biotechnol..

[3] Dufresne A. (2017). Nanocellulose: From Nature to High Performance Tailored Materials.

[4] Farahbakhsh N., Roodposhti P.S., Ayoub A., Venditti R.A., Jur J.S. (2015). Melt extrusion of polyethylene nanocomposites reinforced with nanofibrillated cellulose from cotton and wood sources. J. Appl. Polym. Sci..

[5] Kiziltas A., Nazari B., Kiziltas E.E., Gardner D.J., Han Y., Rushing T.S. (2016). Cellulose nanofiber-polyethylene nanocomposites modified by polyvinyl alcohol. J. Appl. Polym. Sci..

[6] Maia T.H.S., Larocca N.M., Beatrice C.A.G., de Menezes A.J., de Freitas Siqueira G., Pessan L.A., Dufresne A., Franca M.P., de Almeida Lucas A. (2017). Polyethylene cellulose nanofibrils nanocomposites. Carbohydr. Polym..

[7] Yang H.-S., Gardner D.J., Nader J.W. (2011). Characteristic impact resistance model analysis of cellulose nanofibril-filled polypropylene composites. Compos. Part A Appl. Sci. Manuf..

[8] Peng Y., Gallegos S.A., Gardner D.J., Han Y., Cai Z. (2016). Maleic anhydride polypropylene modified cellulose nanofibril polypropylene nanocomposites with enhanced impact strength. Polym. Compos..

[9] Qu P., Gao Y., Wu G., Zhang L. (2010). Nanocomposites of poly (lactic acid) reinforced with cellulose nanofibrils. BioResources.

[10] Wang T., Drzal L.T. (2012). Cellulose-nanofiber-reinforced poly (lactic acid) composites prepared by a water-based approach. ACS Appl. Mater. Interfaces.

[11] Silva M., Sanches A., Medeiros E., Mattoso L., McMahan C., Malmonge J. (2014). Nanocomposites of natural rubber and polyaniline-modified cellulose nanofibrils. J. Therm. Anal. Calorim..

[12] Zhang C., Zhai T., Sabo R., Clemons C., Dan Y., Turng L.-S. (2014). Reinforcing natural rubber with cellulose nanofibrils extracted from bleached eucalyptus kraft pulp. J. Biobased Mater. Bioenergy.

[13] Yano H., Sugiyama J., Nakagaito A.N., Nogi M., Matsuura T., Hikita M., Handa K. (2005). Optically transparent composites reinforced with networks of bacterial nanofibers. Adv. Mater..

[14] Novak B.M. (1993). Hybrid nanocomposite materials—between inorganic glasses and organic polymers. Adv. Mater..

[15] Dufresne A. (2013). Nanocellulose: A new ageless bionanomaterial. Mater. Today.

[16] Qing Y., Sabo R., Wu Y., Zhu J., Cai Z. (2015). Self-assembled optically transparent cellulose nanofibril films: Effect of nanofibril morphology and drying procedure. Cellulose.

[17] Li S., Lee P.S. (2017). Development and applications of transparent conductive nanocellulose paper. Sci. Technol. Adv. Mater..

[18] Iwamoto S., Nakagaito A.N., Yano H., Nogi M. (2005). Optically transparent composites reinforced with plant fiber-based nanofibers. Appl. Phys. A.

[19] Nogi M., Iwamoto S., Nakagaito A.N., Yano H. (2009). Optically transparent nanofiber paper. Adv. Mater..

[20] Zhu H., Fang Z., Preston C., Li Y., Hu L. (2014). Transparent paper: Fabrications, properties, and device applications. Energy Environ. Sci..

[21] Biswas S.K., Sano H., Shams M.I., Yano H. (2017). Three-dimensional-moldable nanofiber-reinforced transparent composites with a hierarchically self-assembled “reverse” nacre-like architecture. ACS Appl. Mater. Interfaces.

[22] Zhao M., Ansari F., Takeuchi M., Shimizu M., Saito T., Berglund L., Isogai A. (2018). Nematic structuring of transparent and multifunctional nanocellulose papers. Nanoscale Horiz..

[23] Shimizu M., Saito T., Fukuzumi H., Isogai A. (2014). Hydrophobic, ductile, and transparent nanocellulose films with quaternary alkylammonium carboxylates on nanofibril surfaces. Biomacromolecules.

[24] Shimazaki Y., Miyazaki Y., Takezawa Y., Nogi M., Abe K., Ifuku S., Yano H. (2007). Excellent thermal conductivity of transparent cellulose nanofiber/epoxy resin nanocomposites. Biomacromolecules.

[25] Ansari F., Galland S., Johansson M., Plummer C.J., Berglund L.A. (2014). Cellulose nanofiber network for moisture stable, strong and ductile biocomposites and increased epoxy curing rate. Compos. Part A Appl. Sci. Manuf..

[26] Qing Y., Cai Z., Wu Y., Yao C., Wu Q., Li X. (2015). Facile preparation of optically transparent and hydrophobic cellulose nanofibril composite films. Ind. Crops Prod..

[27] Mi H., Liu C.-H., Chang T.-H., Seo J.-H., Zhang H., Cho S.J., Behdad N., Ma Z., Yao C., Cai Z. (2016). Characterizations of biodegradable epoxy-coated cellulose nanofibrils (CNF) thin film for flexible microwave applications. Cellulose.

[28] Im W., Lee S., Park H., Lee H.L., Youn H.J. (2016). Characteristics of cellulose nanofibrils by carboxymethylation pretreatment: Effect of the carboxyl contents. J Korea TAPPI.

[29] Sood Y.V., Tyagi R., Tyagi S., Pande P.C., Tondon R. (2010). Surface charge of different paper making raw materials and its influence on paper properties. J. Sci. Ind. Res..

[30] Zhu H., Parvinian S., Preston C., Vaaland O., Ruan Z., Hu L. (2013). Transparent nanopaper with tailored optical properties. Nanoscale.

[31] Bohren C.F., Huffman D.R. (2004). Absorption and Scattering of Light by Small Particles.

[32] Saba N., Mohammad F., Pervaiz M., Jawaid M., Alothman O.Y., Sain M. (2017). Mechanical, morphological and structural properties of cellulose nanofibers reinforced epoxy composites. Int. J. Biol. Macromol..

